# Transactions of the College of Physicians of Philadelphia

**Published:** 1875-11

**Authors:** 


### Books Reviewed.

*Transactions of the College of Physicians of Philadelphia.* Third Series, Vol I. Philadelphia: Lindsay & Blakiston, 1875.

This volume of the transactions of the College of Physicians contains the papers read before that body from April, 1874, to June, 1875, inclusive. The papers are all of them of an interesting and valuable character, and do credit to the venerable body before which they were presented.

They are twelve in number, and are upon the following subjects: Report of an autopsy upon the bodies of the Siamese Twins; by Harrison Allen, M. D. Case of Adenoid (Hodgkins') Disease; by James H. Huthinson, M. D. Case of Fracture of the Neck of the Scapula; by John Ashhurst, Jr., M. D. On a New Operation for certain cases of Cleft Palate and Bifid Uvula; by William S. Forbes, M. D. On the Operative and Conservative Surgery of the Larger Joints; Excision of the Elbow; by John Ashhurst, Jr., M. D. Experiments on the Lanryneal nerves and nerves of Respiration, in a criminal executed by hanging; by W. W. Keen, M. D. On the use of Nitrite of Amyl in various forms of Spasm, and on its value as an aid to diagnosis; by S. Weir Mitchell, M. D. Case of Acute-Tetauns treat by inhalations of Nitrite of Amyl; by William S. Forbes, M. D. Remarks on Diabetes Insipidus and its treatment by Ergot; by J. N. Da-Costa, M. D. Report on the Surgical Consideration in regard to the propriety of an operaticn for the separation of the Siamese Twins, deduced from the Autopsy; by Wm. H. Pancoast, M. D. Case of Encysted Dropsy of the Peritoneum, in which Supperation had occurred and Adbominal Section was performed, with recovery; by J. Ewing Mears, M. D. Remarks on the preceding paper; by William. Pepper, M. D. Quinia as a Stimulant to the Pregnant Uteıus; by Albert H. Smith, M. D.

The foregoing papers are illustrated by two Chromo-Lithographs, which illustrate Prof. Pancoast's paper, and thirty wood-cuts, nineteen of which illustrate Prof. Allen's article on the Siamese Twins.

The paper by Prof. Pancoast, on the Surgical Anatomy of the Siamese Twins, is a careful inquiry into the propriety of an operation for their separation. From the anatomical observations made, he arrives at the conclusion that no operation could have been made during their adult life, for the purpose of separating them, and their lives preserved, but that it would have been judicious surgery, upon the death of Chang, to have at once applied a strong ligature around the band, as far as possible from Eng, and then have cut through the band between the ligature and the body of Chang. He regards the question of the success of an operation in the childhood of the twins as problematical ; but thinks it would have been the part of wisdom and humanity to have made the effort, using all the precautions employed by Dr. Fatio, in his case in 1869, with such additional ones as might have been suggested.

Several of the other essays demand special mention, but space will not allow the notice which they deserve.

